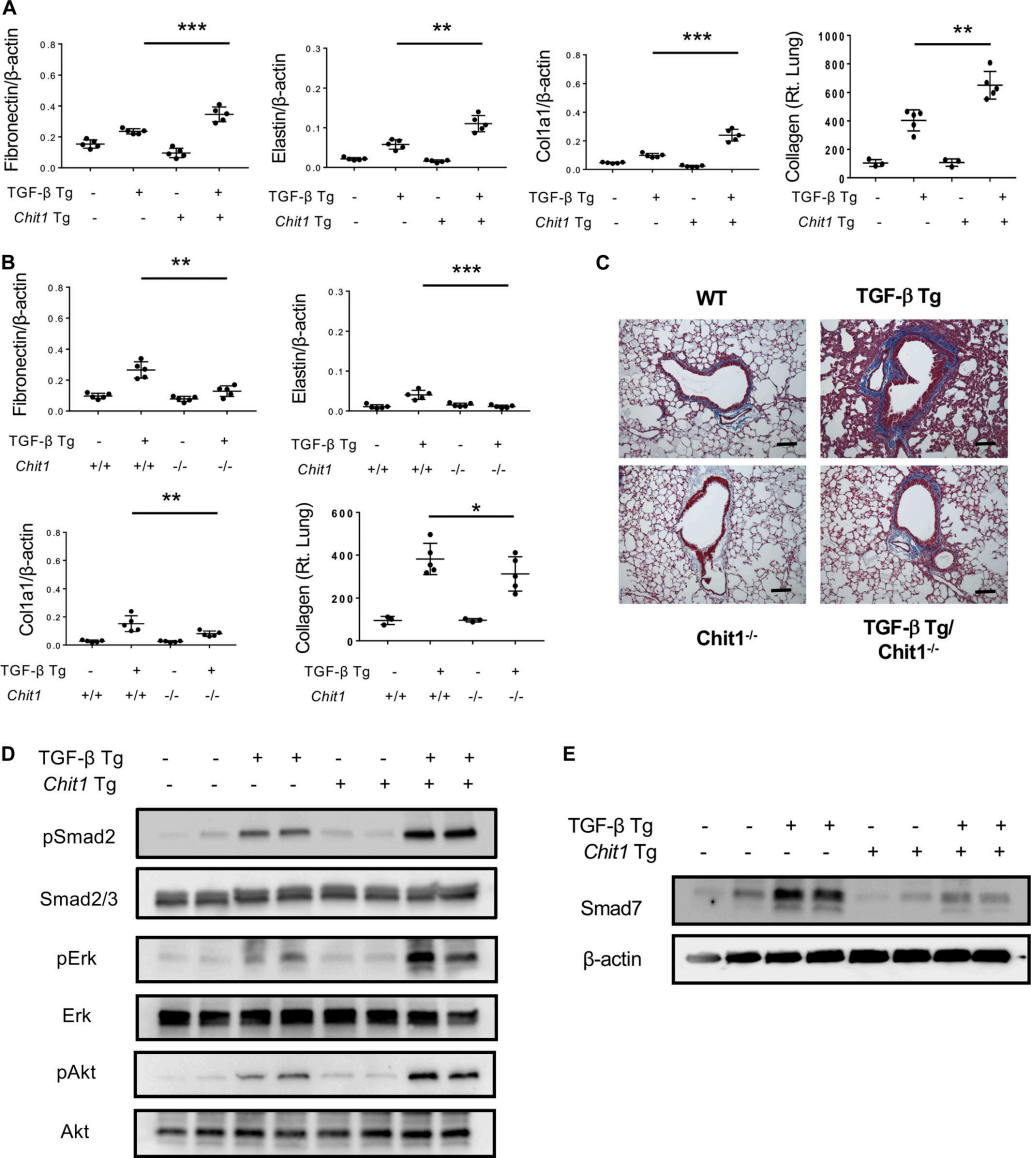# Correction: Chitinase 1 regulates pulmonary fibrosis by modulating TGF-β/SMAD7 pathway via TGFBRAP1 and FOXO3

**DOI:** 10.26508/lsa.202302065

**Published:** 2023-04-10

**Authors:** Chang-Min Lee, Chuan-Hua He, Jin Wook Park, Jae Hyun Lee, Suchitra Kamle, Bing Ma, Bedia Akosman, Roberto Cotez, Emily Chen, Yang Zhou, Erica L Herzog, Changwan Ryu, Xueyan Peng, Ivan O Rosas, Sergio Poli, Carol Feghali Bostwick, Augustine M Choi, Jack A Elias, Chun Geun Lee

**Affiliations:** 1 Molecular Microbiology and Immunology, Brown University , Providence, RI, USA; 2 Department of Internal Medicine, Yonsei University College of Medicine, Seoul, South Korea; 3 Section of Pulmonary, Critical Care, and Sleep Medicine, Department of Internal Medicine, Yale School of Medicine, New Haven, CT, USA; 4 Brigham and Women’s Hospital, Boston, MA, USA; 5 Department of Medicine, College of Medicine, Medical University of South Carolina, Charleston, SC, USA; 6 Weill Cornell Medicine Pulmonary and Critical Care Medicine, New York, NY, USA; 7 Division of Medicine and Biological Sciences, Brown University , Warren Alpert School of Medicine, Providence, RI, USA

## Abstract

Chitinase 1 (CHIT1) plays a role in the pathogenesis of pulmonary fibrosis by modulating canonical and noncanonical TGF-β signaling via interaction with TGFBRAP1 and FOXO3. These findings highlight the CHIT1/SMAD7 axis as a potential biomarker and therapeutic target of pulmonary fibrosis.

Article: Lee C-M, He C-H, Park JW, Lee JH, Kamle S, Ma B, Akosman B, Cotez R, Chen E, Zhou Y, Herzog EL, Ryu C, Peng X, Rosas IO, Poli S, Bostwick CF, Choi AM, Elias JA, Lee CG (2019 May 13) Chitinase 1 regulates pulmonary fibrosis by modulating TGF-β/SMAD7 pathway via TGFBRAP1 and FOXO3. Life Sci Alliance 2(3): e201900350. doi: 10.26508/lsa.201900350. PMID: 31085559.

The authors identified that there were two inadvertent mistakes that led to the duplication of some of the blots that made up the panels in Fig 2D. Specifically, pSmad2 was duplicated with pAkt and Smad2/3 was duplicated with Erk due to the impressive similarities of the expression patterns between these blots. The similarities also caused the omission of the original blots of pSmad2 and Smad2/3. The corrected Fig 2D can be seen below. It is very important to point out that the minor errors in gel usage do not change the scientific conclusions drawn from Fig 2D or the overall results of the study.

Editor’s note: The authors requested the publication of a corrigendum promptly when the error was first noted and the delay in publishing the corrigendum was not due to any fault or delay on the part of the authors of the article.

**Figure:**